# Aerated Waters

**Published:** 1899-11-11

**Authors:** 


### AERATED WATERS.

### THE HAYWARD-TYLER EXHIBITION.

Last week Messrs. Hayward-Tyler and Co. gave an exhibition of their aërated water machinery at their rooms, 90 and 92, Whitecross Street, E.C. The machines are of various sizes and are chiefly of three kinds: The gasometers, or gas holders, the mixing, and the bottling machines; the novelty of the exhibition being the steam power screw-stopper filling machine. This is a compact little machine filling seventy bottles an hour. It is fed by one operator, who places the bottle, with the stopper screwed in, into position. This is enclosed by a strong shield which closes upon the bottle automatically, and which is a safeguard in case of the bottle exploding under the pressure of the gas. The machine unscrews the stopper, fills the bottle with aërated water, or, if desired, syrup and aërated water, and replaces the screw-stopper, throwing the filled bottle into a shoot, whence it is taken and packed ready for use. By an ingenious arrangement the bottle itself presses upon and opens the valve supplying the aërated water. As the pressure is necessary to open the valve it remains shut if the bottle is not in position, consequently there is no waste of either gas or water. The price of this machine is £46.

There is a considerable demand for aërated water supplied in corked bottles. As yet, it is certainly superior to that bottled by any of the patent stoppers, but it is more expensive. Macdonnell's patent automatic bottling and corking machine, therefore, attracted a large share of attention. By it the corks are compressed from the sides and slipped into the bottle neck without crushing, bruising, or turning up. The price is from £60 for the single bottle machine, which fills and corks sixty dozen an hour, requiring the services of one attendant only. The corks are secured by Howard's patent wiring machine. Two strands of wire are carried over the cork and one round each side of the neck. The four are twisted together on either side, a small loop being left on the one to open it by. By simply untwisting this loop the whole of the wiring is detached from the bottle, leaving both the cork and neck free. The price is from £28 15s.

Very useful and efficient, too, are the syphon bottling machines which were on view. The price was from £6. Sidney Munckton's syphon-top cleaner and polisher (price £8 10s.) is also a vast time saver where many syphons have to be refilled. Messrs. Hayward-Tyler have furnished many asylums and large institutions lately with their machines, whilst the London, St. George's, and the Brompton Consumption Hospitals, amongst others, have effected considerable economies by making their own aërated waters with machines provided by this firm.

